# Psychometric Evaluation of a Tablet-Based Tool to Detect Mild Cognitive Impairment in Older Adults: Mixed Methods Study

**DOI:** 10.2196/56883

**Published:** 2024-04-19

**Authors:** Josephine McMurray, AnneMarie Levy, Wei Pang, Paul Holyoke

**Affiliations:** 1 Lazaridis School of Business & Economics Wilfrid Laurier University Brantford, ON Canada; 2 Health Studies, Faculty of Human and Social Sciences Wilfrid Laurier University Brantford, ON Canada; 3 Biomedical Informatics & Data Science Yale University New Haven, CT United States; 4 SE Research Centre Markham, ON Canada

**Keywords:** cognitive dysfunction, dementia neuropsychological tests, evaluation study, technology, aged, mobile phone

## Abstract

**Background:**

With the rapid aging of the global population, the prevalence of mild cognitive impairment (MCI) and dementia is anticipated to surge worldwide. MCI serves as an intermediary stage between normal aging and dementia, necessitating more sensitive and effective screening tools for early identification and intervention. The BrainFx SCREEN is a novel digital tool designed to assess cognitive impairment. This study evaluated its efficacy as a screening tool for MCI in primary care settings, particularly in the context of an aging population and the growing integration of digital health solutions.

**Objective:**

The primary objective was to assess the validity, reliability, and applicability of the BrainFx SCREEN (hereafter, the SCREEN) for MCI screening in a primary care context. We conducted an exploratory study comparing the SCREEN with an established screening tool, the Quick Mild Cognitive Impairment (Qmci) screen.

**Methods:**

A concurrent mixed methods, prospective study using a quasi-experimental design was conducted with 147 participants from 5 primary care Family Health Teams (FHTs; characterized by multidisciplinary practice and capitated funding) across southwestern Ontario, Canada. Participants included health care practitioners, patients, and FHT administrative executives. Individuals aged ≥55 years with no history of MCI or diagnosis of dementia rostered in a participating FHT were eligible to participate. Participants were screened using both the SCREEN and Qmci. The study also incorporated the Geriatric Anxiety Scale–10 to assess general anxiety levels at each cognitive screening. The SCREEN’s scoring was compared against that of the Qmci and the clinical judgment of health care professionals. Statistical analyses included sensitivity, specificity, internal consistency, and test-retest reliability assessments.

**Results:**

The study found that the SCREEN’s longer administration time and complex scoring algorithm, which is proprietary and unavailable for independent analysis, presented challenges. Its internal consistency, indicated by a Cronbach α of 0.63, was below the acceptable threshold. The test-retest reliability also showed limitations, with moderate intraclass correlation coefficient (0.54) and inadequate κ (0.15) values. Sensitivity and specificity were consistent (63.25% and 74.07%, respectively) between cross-tabulation and discrepant analysis. In addition, the study faced limitations due to its demographic skew (96/147, 65.3% female, well-educated participants), the absence of a comprehensive *gold standard* for MCI diagnosis, and financial constraints limiting the inclusion of confirmatory neuropsychological testing.

**Conclusions:**

The SCREEN, in its current form, does not meet the necessary criteria for an optimal MCI screening tool in primary care settings, primarily due to its longer administration time and lower reliability. As the number of digital health technologies increases and evolves, further testing and refinement of tools such as the SCREEN are essential to ensure their efficacy and reliability in real-world clinical settings. This study advocates for continued research in this rapidly advancing field to better serve the aging population.

**International Registered Report Identifier (IRRID):**

RR2-10.2196/25520

## Introduction

### Background

Mild cognitive impairment (MCI) is a syndrome characterized by a slight but noticeable and measurable deterioration in cognitive abilities, predominantly memory and thinking skills, that is greater than expected for an individual’s age and educational level [1,2]. The functional impairments associated with MCI are subtle and often impair instrumental activities of daily living (ADL). Instrumental ADL include everyday tasks such as managing finances, cooking, shopping, or taking regularly prescribed medications and are considered more complex than ADL such as bathing, dressing, and toileting [3,4]. In cases in which memory impairment is the primary indicator of the disease, MCI is classified as amnesic MCI and when significant impairment of non–memory-related cognitive domains such as visual-spatial or executive functioning is dominant, MCI is classified as nonamnesic [5].

Cognitive decline, more so than cancer and cardiovascular disease, poses a substantial threat to an individual’s ability to live independently or at home with family caregivers [6]. The Centers for Disease Control and Prevention reports that 1 in 8 adults aged ≥60 years experiences memory loss and confusion, with 35% reporting functional difficulties with basic ADL [7]. The American Academy of Neurology estimates that the prevalence of MCI ranges from 13.4% to 42% in people aged ≥65 years [8], and a 2023 meta-analysis that included 233 studies and 676,974 participants aged ≥50 years estimated that the overall global prevalence of MCI is 19.7% [9]. Once diagnosed, the prognosis for MCI is variable, whereby the impairment may be reversible; the rate of decline may plateau; or it may progressively worsen and, in some cases, may be a prodromal stage to dementia [10-12]. While estimates vary based on sample (community vs clinical), annual rates of conversion from MCI to dementia range from 5% to 24% [11,12], and those who present with multiple domains of cognitive impairment are at higher risk of conversion [5].

The risk of developing MCI rises with age, and while there are no drug treatments for MCI, nonpharmacologic interventions may improve cognitive function, alleviate the burden on caregivers, and potentially delay institutionalization should MCI progress to dementia [13]. To overcome the challenges of early diagnosis, which currently depends on self-detection, family observation, or health care provider (HCP) recognition of symptoms, screening high-risk groups for MCI or dementia is suggested as a solution [13]. However, the Canadian Task Force on Preventive Health Care recommends against screening adults aged ≥65 years due to a lack of meaningful evidence from randomized controlled trials and the high false-positive rate [14-16]. The main objective of a screening test is to reduce morbidity or mortality in at-risk populations through early detection and intervention, with the anticipated benefits outweighing potential harms. Using brief screening tools in primary care might improve MCI case detection, allowing patients and families to address reversible causes, make lifestyle changes, and access disease-modifying treatments [17].

There is no agreement among experts as to which tests or groups of tests are most predictive of MCI [16], and the gold standard approach uses a combination of positive results from neuropsychological assessments, laboratory tests, and neuroimaging to infer a diagnosis [8,18]. The clinical heterogeneity of MCI complicates its diagnosis because it influences not only memory and thinking abilities but also mood, behavior, emotional regulation, and sensorimotor abilities, and patients may present with any combination of symptoms with varying rates of onset and decline [4,8]. For this reason, a collaborative approach between general practitioners and specialists (eg, geriatricians and neurologists) is often required to be confident in the diagnosis of MCI [8,19,20].

In Canada, diagnosis often begins with screening for cognitive impairment followed by referral for additional testing; this process takes, on average, 5 months [20]. The *current usual practice* screening tools for MCI are the Mini-Mental State Examination (MMSE) [21,22] and the Montreal Cognitive Assessment (MoCA) 8.1 [3]. Both are paper-and-pencil screens administered in 10 to 15 minutes, scored out of 30, and validated as MCI screening tools across diverse clinical samples [23,24]. Universally, the MMSE is most often used to screen for MCI [20,25] and consists of 20 items that measure orientation, immediate and delayed recall, attention and calculation, visual-spatial skills, verbal fluency, and writing. The MoCA 8.1 was developed to improve on the MMSE’s ability to detect early signs of MCI, placing greater emphasis on evaluating executive function as well as language, memory, visual-spatial skills, abstraction, attention, concentration, and orientation across 30 items [24,26]. Scores of <24 on the MMSE or ≤25 on the MoCA 8.1 signal probable MCI [21,27]. Lower cutoff scores for both screens have been recommended to address evidence that they lack specificity to detect mild and early cases of MCI [4,28-31]. The clinical efficacy of both screens for tracking change in cognition over time is limited as they are also subject to practice effects with repeated administration [32].

Novel screening tools, including the Quick Mild Cognitive Impairment (Qmci) screen, have been developed with the goal of improving the accuracy of detecting MCI [33,34]. The Qmci is a sensitive and specific tool that differentiates normal cognition from MCI and dementia and is more accurate at differentiating MCI from controls than either the MoCA 8.1 (Qmci area under the curve=0.97 vs MoCA 8.1 area under the curve=0.92) [25,35] or the Short MMSE [33,36]. It also demonstrates high test-retest reliability (intraclass correlation coefficient [ICC]=0.88) [37] and is clinically useful as a rapid screen for MCI as the Qmci mean is 4.5 (SD 1.3) minutes versus 9.5 (SD 2.8) minutes for the MoCA 8.1 [25].

The COVID-19 pandemic and the necessary shift to virtual health care accelerated the use of digital assessment tools, including MCI screening tools such as the electronic MoCA 8.1 [38,39], and the increased use and adoption of technology (eg, smartphones and tablets) by older adults suggests that a lack of proficiency with technology may not be a barrier to the use of such assessment tools [40,41]. BrainFx is a for-profit firm that creates proprietary software designed to assess cognition and changes in neurofunction that may be caused by neurodegenerative diseases (eg, MCI or dementia), stroke, concussions, or mental illness using ecologically relevant tasks (eg, prioritizing daily schedules and route finding on a map) [42]. Their assessments are administered via a tablet and stylus. The BrainFx 360 performance assessment (referred to hereafter as the 360) is a 90-minute digitally administered test that was designed to assess cognitive, physical, and psychosocial areas of neurofunction across 26 cognitive domains using 49 tasks that are timed and scored [42]. The BrainFx SCREEN (referred to hereafter as the SCREEN) is a short digital version of the 360 that includes 7 of the cognitive domains included in the 360, is estimated to take approximately 10 to 15 minutes to complete, and was designed to screen for early detection of cognitive impairment [43,44]. Upon completion of any BrainFx assessment, the results of the 360 or SCREEN are added to the BrainFx Living Brain Bank (LBB), which is an electronic database that stores all completed 360 and SCREEN assessments and is maintained by BrainFx. An electronic report is generated by BrainFx comparing an individual’s results to those of others collected and stored in the LBB. Normative data from the LBB are used to evaluate and compare an individual’s results.

The 360 has been used in clinical settings to assess neurofunction among youth [45] and anecdotally in other rehabilitation settings (T Milner, personal communication, May 2018). To date, research on the 360 indicates that it has been validated in healthy young adults (mean age 22.9, SD 2.4 years) and that the overall test-retest reliability of the tool is high (ICC=0.85) [42]. However, only 2 of the 7 tasks selected to be included in the SCREEN produced reliability coefficients of >0.70 (visual-spatial and problem-solving abilities) [42]. Jones et al [43] explored the acceptability and perceived usability of the SCREEN with a small sample (N=21) of Canadian Armed Forces veterans living with posttraumatic stress disorder. A structural equation model based on the Unified Theory of Acceptance and Use of Technology suggested that behavioral intent to use the SCREEN was predicted by facilitating conditions such as guidance during the test and appropriate resources to complete the test [43]. However, the validity, reliability, and sensitivity of the SCREEN for detecting cognitive impairment have not been tested.

### Objectives

McMurray et al [44] designed a protocol to assess the validity, reliability, and sensitivity of the SCREEN for detecting early signs of MCI in asymptomatic adults aged ≥55 years in a primary care setting (5 Family Health Teams [FHTs]). The protocol also used a series of semistructured interviews and surveys guided by the fit between individuals, task, technology, and environment framework [46], a health-specific model derived from the Task-Technology Fit model by Goodhue and Thompson [47], to explore the SCREEN’s acceptability and use by HCPs and patients in primary care settings (manuscript in preparation). This study is a psychometric evaluation of the SCREEN’s validity, reliability, and sensitivity for detecting MCI in asymptomatic adults aged ≥55 years in primary care settings.

## Methods

### Study Location, Design, and Data Collection

This was a concurrent, mixed methods, prospective study using a quasi-experimental design. Participants were recruited from 5 primary care FHTs (characterized by multidisciplinary practice and capitated funding) across southwestern Ontario, Canada. FHTs that used a registered occupational therapist on staff were eligible to participate in the study, and participating FHTs received a nominal compensatory payment for the time the HCPs spent in training; collecting data for the study; administering the SCREEN, Qmci, and Geriatric Anxiety Scale–10 (GAS-10); and communicating with the research team. A multipronged recruitment approach was used [44]. A designated occupational therapist at each location was provided with training and equipment to recruit participants, administer assessment tools, and submit collected data to the research team.

The research protocol describing the methods of both the quantitative and qualitative arms of the study is published elsewhere [44].

### Ethical Considerations

This study was approved by the Wilfrid Laurier University Research Ethics Board (ORE 5820) and was reviewed and approved by each FHT. Participants (HCPs, patients, and administrative executives) read and signed an information and informed consent package in advance of taking part in the study. We complied with recommendations for obtaining informed consent and conducting qualitative interviews with persons with dementia when recruiting patients who may be affected by neurocognitive diseases [48-50]. In addition, at the end of each SCREEN assessment, patients were required to provide their consent (electronic signature) to contribute their anonymized scores to the database of SCREEN results maintained by BrainFx. Upon enrolling in the study, participants were assigned a unique identification number that was used in place of their name on all study documentation to anonymize the data and preserve their confidentiality. A master list matching participant names with their unique identification number was stored in a password-protected file by the administering HCP and principal investigator on the research team. The FHTs received a nominal compensatory payment to account for their HCPs’ time spent administering the SCREEN, collecting data for the study, and communicating with the research team. However, the individual HCPs who volunteered to participate and the patient participants were not financially compensated for taking part in the study.

### Participants

Patients who were rostered with the FHT, were aged ≥55 years, and had no history of MCI or dementia diagnoses to better capture the population at risk of early signs of cognitive impairment were eligible to participate [51,52]. It was necessary for the participants to be rostered with the FHTs to ensure that the HCPs could access their electronic medical record to confirm eligibility and record the testing sessions and results and to ensure that there was a responsible physician for referral if indicated. As the SCREEN is administered using a tablet, participants had to be able to read and think in English and discern color, have adequate hearing and vision to interact with the administering HCP, read 12-point font on the tablet, and have adequate hand and arm function to manipulate and hold the tablet. The exclusion criteria used in the study included colorblindness and any disability that might impair the individual’s ability to hold and interact with the tablet. Prospective participants were also excluded based on a diagnosis of conditions that may result in MCI or dementia-like symptoms, including major depression that required hospitalization, psychiatric disorders (eg, schizophrenia and bipolar disorder), psychopathology, epilepsy, substance use disorders, or sleep apnea (without the use of a continuous positive airway pressure machine) [52]. Patients were required to complete a minimum of 2 screening sessions spaced 3 months apart to participate in the study and, depending on when they enrolled to participate, could complete a maximum of 4 screening sessions over a year.

### Data Collection Instruments

#### GAS-10 Instrument

A standardized protocol was used to collect demographic data, randomly administer the SCREEN and the Qmci (a validated screening tool for MCI), and administer the GAS-10 immediately before and after the completion of the first MCI screen at each visit [44]. This was to assess participants’ general anxiety as it related to screening for cognitive impairment at the time of the assessment, any change in subjective ratings after completion of the first MCI screen, and change in anxiety between appointments. The GAS-10 is a 10-item, self-report screen for anxiety in older adults [53] developed for rapid screening of anxiety in clinical settings (the GAS-10 is the short form of the full 30-item Geriatric Anxiety Scale [GAS]) [54]. While 3 subscales are identified, the GAS is reported to be a unidimensional scale that assesses general anxiety [55,56]. Validation of the GAS-10 suggests that it is optimal for assessing average to moderate levels of anxiety in older adults, with subscale scores that are highly and positively correlated with the GAS and high internal consistency [53]. Participants were asked to use a 4-point Likert scale (0=*not at all*, 1=*sometimes*, 2=*most of the time*, and 3=*all of the time*) to rate how often they had experienced each symptom over the previous week, including on the day the test was administered [54]. The GAS-10 has a maximum score of 30, with higher scores indicating higher levels of anxiety [53,54,57].

#### The SCREEN

HCPs completed the required training to become certified BrainFx SCREEN administrators before the start of the study. To this end, HCPs completed a web-based training program (developed and administered through the BrainFx website) that included 3 self-directed training modules. For the purpose of the study, they also participated in 1 half-day in-person training session conducted by a certified BrainFx administrator (T Milner, BrainFx chief executive officer) at one of the participating FHT locations. The SCREEN (version 0.5; beta) was administered on a tablet (ASUS ZenPad 10.1” IPS WXGA display, 1920 × 1200, powered by a quad-core 1.5 GHz, 64-bit MediaTek MTK 8163A processor with 2 GB RAM and 16-GB storage). The tablet came with a tablet stand for optional use and a dedicated stylus that is recommended for completion of a subset of questions. At the start of the study, HCPs were provided with identical tablets preloaded with the SCREEN software for use in the study. The 7 tasks on the SCREEN are summarized in Table 1 and were taken directly from the 360 based on a clustering and regression analysis of LBB records in 2016 (N=188) [58]. A detailed description of the study and SCREEN administration procedures was published by McMurray et al [44].

**Table 1:** Summary of the 7 SCREEN tasks and the length of time allotted to complete them.

Task	Description	Time to complete
Abstract reasoning	20 everyday items are displayed, and the patient touches the item on the screen and slides each item, one at a time, into 1 of 5 categories to which they best belong while timed.	90 seconds
Constructive ability	2 rounds of a photo are displayed, and it is broken into 9 pieces. The patient touches the pieces and slides each piece into a grid to reassemble the picture while timed.	90 seconds
Prioritizing	5 everyday activities or tasks are presented, the patient is told what time of day it is (eg, 7 PM), and they touch the screen and slide each item to prioritize the order in which the activities or tasks should be completed.	60 seconds
Numerical problem-solving	10 math questions, with 1- or 2-digit answers, are presented for patient response using a numerical pad (+, –, ×, and /) while timed.	90 seconds
Visual-spatial ability	2 rounds of the patient selecting (by touch) into which shape a word fits best while timed.	30 seconds
Divided attention	The patient watches a pot on the stove about to boil over (denoted by boiling water and a red signal) and must touch the pot and move it to the sink to dump out the water while also touching the screen to match as many objects as they can within the kitchen scene.	90 seconds
Route finding	In round 1, the patient traces the most efficient route between 2 locations while timed. In round 2, the patient traces the most efficient route between 2 locations but is instructed to make 2 stops on the way while timed.	90 seconds

An *activity score* is generated for each of the 7 tasks on the SCREEN. It is computed based on a combination of the accuracy of the participant’s response and the processing speed (time in seconds) that it takes to complete the task. The relative contribution of accuracy and processing speed to the final activity score for each task is proprietary to BrainFx and unknown to the research team. The participant’s activity score is compared to the mean activity score for the same task at the time of testing in the LBB. The mean activity score from the LBB may be based on the global reference population (ie, all available SCREEN results in the LBB), or the administering HCP may select a specific reference population by filtering according to factors including but not limited to age, sex, or diagnosis. If the participant’s activity score is >1 SD below the LBB activity score mean for that task, it is labeled as an *area of challenge*. Each of the 7 tasks on the SCREEN are evaluated independently of each other, producing a report with 7 activity scores showing the participant’s score, the LBB mean score, and the SD. The report also provides an overall performance and processing speed score. The overall performance score is an average of all 7 activity scores; however, the way in which the overall processing speed score is generated remains proprietary to BrainFx and unknown to the research team. Both the overall performance and processing speed scores are similarly evaluated against the LBB and identified as an area of challenge using the criteria described previously. For the purpose of this study, participants’ mean activity scores on the SCREEN were compared to the results of people aged ≥55 years in the LBB.

#### The Qmci

The Qmci evaluated 6 cognitive domains: orientation (10 points), registration (5 points), clock drawing (15 points), delayed recall (20 points), verbal fluency (20 points), and logical memory (30 points) [59]. Administering HCPs scored the text manually, with each subtest’s points contributing to the overall score out of 100 points, and the cutoff score to distinguish normal cognition from MCI was ≤67/100 [60]. Cutoffs to account for age and education have been validated and are recommended as the Qmci is sensitive to these factors [60]. A 2019 meta-analysis of the diagnostic accuracy of MCI screening tools reported that the sensitivity and specificity of the Qmci for distinguishing MCI from normal cognition is similar to usual standard-of-care tools (eg, the MoCA, Addenbrooke Cognitive Examination–Revised, Consortium to Establish a Registry for Alzheimer’s Disease battery total score, and Sunderland Clock Drawing Test) [61]. The Qmci has also been translated into >15 different languages and has undergone psychometric evaluation across a subset of these languages. While not as broadly adopted as the MoCA 8.1 in Canada, its psychometric properties, administration time, and availability for use suggested that the Qmci was an optimal assessment tool for MCI screening in FHT settings during the study.

### Psychometric Evaluation

#### Overview

To date, the only published psychometric evaluation of any BrainFx tool is by Searles et al [42] in *Athletic Training & Sports Health Care*; it assessed the test-retest reliability of the 360 in 15 healthy adults between the ages of 20 and 25 years. This study evaluated the psychometric properties of the SCREEN and included a statistical analysis of the tool’s internal consistency, construct validity, test-retest reliability, and sensitivity and specificity. McMurray et al [44] provide a detailed description of the data collection procedures for administration of the SCREEN and Qmci completed by participants at each visit.

#### Validity Testing

Face validity was outside the scope of this study but was implied, and assumptions are reported in the Results section. Construct validity, whether the 7 activities that make up the SCREEN were representative of MCI, was assessed through comparison with a substantive body of literature in the domain and through principal component analysis using varimax rotation. Criterion validity measures how closely the SCREEN results corresponded to the results of the Qmci (used here as an “imperfect gold standard” for identifying MCI in older adults) [62]. A BrainFx representative hypothesized that the ecological validity of the SCREEN questions (ie, using tasks that reflect real-world activities to detect early signs of cognitive impairment) [63] makes it a more sensitive tool than other screens (T Milner, personal communication, May 2018) and allows HCPs to equate activity scores on the SCREEN with real-world functional abilities. Criterion validity was explored first using cross-tabulations to calculate the sensitivity and specificity of the SCREEN compared to those of the Qmci. Conventional screens such as the Qmci are scored by taking the sum of correct responses on the screen and a cutoff score derived from normative data to distinguish normal cognition from MCI. The SCREEN used a different method of scoring whereby each of the 7 tasks was scored and evaluated independently of each other and there were no recommended guidelines for distinguishing normal cognition from MCI based on the aggregate areas of challenge identified by the SCREEN. Therefore, to compare the sensitivity and specificity of the SCREEN against those of the Qmci, the results of both screens were coded into a binary format as 1=healthy and 2=unhealthy, where *healthy* denoted no areas of challenge identified through the SCREEN and a Qmci score of ≥67. Conversely, *unhealthy* denoted one or more areas of challenge identified through the SCREEN and a Qmci score of <67.

Criterion validity was further explored using discrepant analysis via a resolver test [44]. Following the administration of the SCREEN and Qmci, screen results were evaluated by the administering HCP. HCPs were instructed to refer the participant for follow-up with their primary care physician if the Qmci result was <67 regardless of whether any areas of challenge were identified on the SCREEN. However, HCPs could use their clinical judgment to refer a participant for physician follow-up based on the results of the SCREEN or the Qmci, and all the referral decisions were charted on the participant’s electronic medical record following each visit and screening. In discrepant analysis, the results of the *imperfect gold standard* [64], as was the role of the Qmci in this study, were compared with the SCREEN results. A resolver test (classified as whether the HCP referred the patient to a physician for follow-up based on their performance on the SCREEN and the Qmci) was used on discordant results [64,65] to determine sensitivity and specificity. To this end, a new variable, *Referral to a Physician for Cognitive Impairment*, was coded as the *true status* (1=no referral; 2=referral was made) and compared to the Qmci as the *imperfect gold standard* (1=healthy; 2=unhealthy).

#### Reliability Testing

The reliability of a screening instrument is its ability to consistently measure an attribute and how well its component measures fit together conceptually. Internal consistency identifies whether the items in a multi-item scale are measuring the same underlying construct; the internal consistency of the SCREEN was assessed using the Cronbach α. Test-retest reliability refers to the ability of a measurement instrument to reproduce results over ≥2 occasions (assuming the underlying conditions have not changed) and was assessed using paired *t* tests (2-tailed), ICC, and the κ coefficient. In this study, participants completed both the SCREEN and the Qmci in the same sitting in a random sequence on at least 2 different occasions spaced 3 months apart (administration procedures are described elsewhere) [44]. In some instances, the screens were administered to the same participant on 4 separate occasions spaced 3 months apart each, and this provided up to 3 separate opportunities to conduct test-retest reliability analyses and investigate the effects of repeated practice. There are no clear guidelines on the optimal time between tests [66,67]; however, Streiner and Kottner [68] and Streiner [69] recommend longer periods between tests (eg, at least 10-14 days) to avoid recall bias, and greater practice effects have been experienced with shorter test-retest intervals [32].

### Statistics

Analysis of the quantitative data was completed using Stata (version 17.0; StataCorp). Assumptions of normality were not violated, so parametric tests were used. Collected data were reported using frequencies and percentages and compared using the chi-square or Fisher exact test as necessary. Continuous data were analyzed for central tendency and variability; categoric data were presented as proportions. Normality was tested using the Shapiro-Wilk test, and nonparametric data were tested using the Mann-Whitney *U* test. A *P* value of .05 was considered statistically significant, with 95% CIs provided where appropriate. We powered the exploratory analysis to validate the SCREEN using an estimated effect size of 12%—understanding that Canadian prevalence rates of MCI were not available [1]—and determined that the study required at least 162 participants. For test-retest reliability, using 90% power and a 5% type-I error rate, a minimum of 67 test results was required.

The time taken for participants to complete the SCREEN was recorded by the HCPs at the time of testing; there were 6 missing HCP records of time to complete the SCREEN. For these 6 cases of missing data, we imputed the mean time to complete the SCREEN by all participants who were tested by that HCP and used this to populate the missing cells [70]. There were 3 cases of missing data related to the SCREEN reports. More specifically, the SCREEN report generated by BrainFx did not include 1 or 2 data points each for the route finding, divided attention, and prioritizing tasks. The clinical notes provided by the HCP at the time of SCREEN administration did not indicate that the participant had not completed those questions, and it was not possible to determine the root cause of the missing data in report generation according to BrainFx (M Milner, personal communication, July 7, 2020). For continuous variables in analyses such as exploratory factor analysis, Cronbach α, and *t* test, missing values were imputed using the mean. However, for the coded healthy and unhealthy categorical variables, values were not imputed.

## Results

Data collection began in January 2019 and was to conclude on May 31, 2020. However, the emergence of the global COVID-19 pandemic resulted in the FHTs and Wilfrid Laurier University prohibiting all in-person research starting on March 16, 2020.

### Participant Demographics

A total of 154 participants were recruited for the study, and 20 (13%) withdrew following their first visit to the FHT. The data of 65% (13/20) of the participants who withdrew were included in the final analysis, and the data of the remaining 35% (7/20) were removed, either due to their explicit request (3/7, 43%) or because technical issues at the time of testing rendered their data unusable (4/7, 57%). These technical issues were related to software issues (eg, any instance in which the patient or HCP interacted with the SCREEN software and followed the instructions provided, the software did not work as expected [ie, objects did not move where they were dragged or tapping on objects failed to highlight the object], and the question could not be completed). After attrition, a total of 147 individuals aged ≥55 years with no previous diagnosis of MCI or dementia participated in the study (Table 2). Of the 147 participants, 71 (48.3%) took part in only 1 round of screening on visit 1 (due to COVID-19 restrictions imposed on in-person research that prevented a second visit). The remaining 51.7% (76/147) of the participants took part in ≥2 rounds of screening across multiple visits (76/147, 51.7% participated in 2 rounds; 22/147, 15% participated in 3 rounds; and 13/147, 8.8% participated in 4 rounds of screening).

**Table 2:** Study participant demographics (highest level of education attained and age; N=147).

Characteristics		Total, n (%)	Female participants (n=96, 65.3%), n (%)	Male participants (n=51, 34.7%), n (%)
**Education**				
	Lower than high school	6 (4.1)	5 (3.4)	1 (0.7)
	High school	45 (30.6)	30 (20.4)	15 (10.2)
	College diploma or certificate	39 (26.5)	27 (18.4)	12 (8.2)
	University degree	34 (23.1)	22 (15)	12 (8.2)
	Postgraduate degree	23 (15.6)	12 (8.2)	11 (7.5)
**Age (years)**				
	55-59	14 (9.5)	10 (6.8)	4 (2.7)
	60-64	17 (11.6)	12 (8.2)	5 (3.4)
	65-69	35 (23.8)	25 (17)	10 (6.8)
	70-74	33 (22.4)	23 (15.6)	10 (6.8)
	75-79	25 (17)	12 (8.2)	13 (8.8)
	80-84	14 (9.5)	9 (6.1)	5 (3.4)
	85-89	9 (6.1)	5 (3.4)	4 (2.7)
	≥90	0 (0)	0 (0)	0 (0)

The sample population was 65.3% (96/147) female (mean 70.2, SD 7.9 years) and 34.7% (51/147) male (mean 72.5, SD 8.1 years), with age ranging from 55 to 88 years; 65.3% (96/147) achieved the equivalent of or higher than a college diploma or certificate (Table 2); and 32.7% (48/147) self-reported living with one or more chronic medical conditions (Table 3). At the time of screening, 73.5% (108/147) of participants were also taking medications with side effects that may include impairments to memory and thinking abilities [71-75]; therefore, medication use was accounted for in a subset of the analyses. Finally, 84.4% (124/147) of participants self-reported regularly using technology (eg, smartphone, laptop, or tablet) with high proficiency. A random sequence generator was used to determine the order for administering the MCI screens; the SCREEN was administered first 51.9% (134/258) of the time.

**Table 3:** Self-reported status of participants’ health with respect to preexisting conditions and chronic disease types (N=147).

Participant health status		Participants, n (%)
**Preexisting conditions**		
	None	99 (67.3)
	Single condition	37 (25.2)
	Comorbidity	7 (4.8)
	Multimorbidity	4 (2.7)
**Chronic disease types reported**		
	Cardiovascular diseases	7 (4.8)
	Chronic respiratory diseases	3 (2)
	Diabetes	5 (3.4)
	Musculoskeletal disorders	10 (6.8)
	Neurological conditions	0 (0)
	Cancer	6 (4.1)
	Mental illnesses	19 (12.9)
	Other (eg, autoimmune or chronic pain)	11 (7.5)

### Validity Testing

#### Construct Validity

Construct validity was assessed through a review of relevant peer-reviewed literature that compared constructs included in the SCREEN with those identified in the literature as 2 of the most sensitive tools for MCI screening: the MoCA 8.1 [76] and the Qmci [25]. Memory, language, and verbal skills are assessed in the MoCA and Qmci but are absent from the SCREEN. Tests of verbal fluency and logical memory have been shown to be particularly sensitive to early cognitive changes [77,78] but are similarly absent from the SCREEN.

Exploratory factor analysis was performed to examine the SCREEN’s ability to reliably measure risk of MCI. The Kaiser-Meyer-Olkin measure yielded a value of 0.79, exceeding the commonly accepted threshold of 0.70, indicating that the sample was adequate for factor analysis. The Bartlett test of sphericity returned a chi-square value of *χ*^2^_21_=167.1 (*P*<.001), confirming the presence of correlations among variables suitable for factor analysis. A principal component analysis revealed 2 components with eigenvalues of >1, cumulatively accounting for 52.12% of the variance, with the first factor alone explaining 37.8%. After the varimax rotation, the 2 factors exhibited distinct patterns of loadings, with the visual-spatial ability factor loading predominantly on the second factor. The SCREEN tasks, except for visual-spatial ability, loaded substantially on the factors (>0.5), suggesting that the SCREEN possesses good convergent validity for assessing the risk of MCI.

#### Criterion Validity

The coding of SCREEN scores into a binary *healthy* and *unhealthy* outcome standardized the dependent variable to allow for criterion testing. Criterion validity was assessed using cross-tabulations and the analysis of confusion matrices and provided insights into the sensitivity and specificity of the SCREEN when compared to the Qmci. Of the 144 cases considered, 20 (13.9%) were true negatives, and 74 (51.4%) were true positives. The SCREEN’s sensitivity, which reflects its capacity to accurately identify healthy individuals (true positives), was 63.25% (74 correct identifications/117 actual positives). The specificity of the test, indicating its ability to accurately identify unhealthy individuals (true negatives), was 74.07% (20 correct identifications/27 actual negatives). Then, sensitivity and specificity were derived using discrepant analysis and a resolver test previously described (whether the HCP referred the participant to a physician following the screens). The results were identical, the estimate of the SCREEN sensitivity was 63.3% (74/117), and the estimate of the specificity was 74% (20/27).

### Reliability Testing

#### Internal Reliability

A Cronbach α=0.70 is acceptable, and at least 0.90 is required for clinical instruments [79]. The estimate of internal consistency for the SCREEN (N=147) was Cronbach α=0.63.

#### Test-Retest Reliability

Test-retest reliability analyses were conducted using ICC for the SCREEN activity scores and the κ coefficient for the healthy and unhealthy classifications. Guidelines for interpretation of the ICC suggest that anything <0.5 indicates poor reliability and anything between 0.5 and 0.75 suggests moderate reliability [80]; the ICC for the SCREEN activity scores was 0.54. With respect to the κ coefficient, a κ value of <0.2 is considered to have no level of agreement, a κ value of 0.21 to 0.39 is considered minimal, a κ value of 0.4 to 0.59 is considered weak agreement, and anything >0.8 suggests strong to almost perfect agreement [81]. The κ coefficient for healthy and unhealthy classifications was 0.15.

### Analysis of the Factors Impacting Healthy and Unhealthy Results

The Spearman rank correlation was used to assess the relationships between participants’ overall activity score on the SCREEN and their total time to complete the SCREEN; age, sex, and self-reported levels of education; technology use; medication use; amount of sleep; and level of anxiety (as measured using the GAS-10) at the time of SCREEN administration. Lower overall activity scores were moderately correlated with being older (*r*_s142_=–0.57; *P*<.001) and increased total time to complete the SCREEN (*r*_s142_=0.49; *P*<.001). There was also a moderate inverse relationship between overall activity score and total time to compete the SCREEN (*r*_s142_=–0.67; *P*<.001) whereby better performance was associated with quicker task completion. There were weak positive associations between overall activity score and increased technology use (*r*_s142_=0.34; *P*<.001) and higher level of education (*r*_s142_=0.21; *P*=.01).

A logistic regression model was used to predict the SCREEN result using data from 144 observations. The model’s predictors explain approximately 21.33% of the variance in the outcome variable. The likelihood ratio test indicates that the model provides a significantly better fit to the data than a model without predictors (*P*<.001).

The SCREEN outcome variable (*healthy* vs *unhealthy*) was associated with the predictor variables sex and total time to complete the SCREEN. More specifically, female participants were more likely to obtain *healthy* SCREEN outcomes (*P*=.007; 95% CI 0.32-2.05). For all participants, the longer it took to complete the SCREEN, the less likely they were to achieve a *healthy* SCREEN outcome (*P*=.002; 95% CI –0.33 to –0.07). Age (*P*=.25; 95% CI –0.09 to 0.02), medication use (*P*=.96; 95% CI –0.9 to 0.94), technology use (*P*=.44; 95% CI –0.28 to 0.65), level of education (*P*=.14; 95% CI –0.09 to 0.64), level of anxiety (*P*=.26; 95% CI –1.13 to 0.3), and hours of sleep (*P*=.08; 95% CI –0.06 to 0.93) were not significant.

### Impact of Practice Effects

The SCREEN was administered approximately 3 months apart, and separate, paired-sample *t* tests were performed to compare SCREEN outcomes between visits 1 and 2 (76/147, 51.7%; Table 4), visits 2 and 3 (22/147, 15%), and visits 3 and 4 (13/147, 8.8%). Declining visits were partially attributable to the early shutdown of data collection due to the COVID-19 pandemic, and therefore, comparisons between visits 2 and 3 or visits 3 and 4 were not reported. Compared to participants’ SCREEN performance on visit 1, their overall mean activity score and overall processing time improved on their second administration of the SCREEN (score: t_75_=–2.86 and *P*=.005; processing time: t_75_=–2.98 and *P*=.004). Even though the 7 task-specific activity scores on the SCREEN also increased between visits 1 and 2, these improvements were not significant, indicating that the difference in overall activity scores was cumulative and not attributable to a specific task (Table 4).

**Table 4:** Change in participants’ SCREEN scores between visits 1 and 2.

SCREEN task	Visit 1 (n=76), mean (SD)	Visit 2 (n=76), mean (SD)	*t* test (*df*)	*P* value
Abstract reasoning	73.51 (1.61)	75.41 (1.87)	–1.04 (75)	.30
Constructive ability	44.15 (2.79)	46.76 (2.58)	–0.91 (75)	.37
Visual-spatial ability	82.22 (2.04)	85.70 (2.0)	–1.19 (75)	.24
Numerical problem-solving	77.82 (1.64)	80.12 (1.71)	–1.27 (75)	.21
Route finding	57.75 (2.64)	56.52 (2.58)	–0.39 (75)	.70
Prioritizing	57.11 (2.1)	55.57 (2.48)	0.55 (75)	.59
Divided attention	56.44 (2.15)	60.04 (2.28)	–1.77 (75)	.08

## Discussion

### Principal Findings

Our study aimed to evaluate the effectiveness and reliability of the BrainFx SCREEN in detecting MCI in primary care settings. The research took place during the COVID-19 pandemic, which influenced the study’s execution and timeline. Despite these challenges, the findings offer valuable insights into cognitive impairment screening.

Brief MCI screening tools help time-strapped primary care physicians determine whether referral for a definitive battery of more time-consuming and expensive tests is warranted. These tools must optimize and balance the need for time efficiency while also being psychometrically valid and easily administered [82]. The importance of brevity is determined by a number of factors, including the clinical setting. Screens that can be completed in approximately ≤5 minutes [13] are recommended for faster-paced clinical settings (eg, emergency rooms and preoperative screens), whereas those that can be completed in 5 to 10 minutes or less are better suited to primary care settings [82-84]. Identifying affordable, psychometrically tested screening tests for MCI that integrate into clinical workflows and are easy to consistently administer and complete may help with the following:

1. Initiating appropriate diagnostic tests for signs and symptoms at an earlier stage
2. Normalizing and destigmatizing cognitive testing for older adults
3. Expediting referrals
4. Allowing for timely access to programs and services that can support aging in place or delay institutionalization
5. Reducing risk
6. Improving the psychosocial well-being of patients and their care partners by increasing access to information and resources that aid with future planning and decision-making [85,86]

Various cognitive tests are commonly used for detecting MCI. These include the Addenbrook Cognitive Examination–Revised, Consortium to Establish a Registry for Alzheimer’s Disease, Sunderland Clock Drawing Test, Informant Questionnaire on Cognitive Decline in the Elderly, Memory Alternation Test, MMSE, MoCA 8.1, and Qmci [61,87]. The Addenbrook Cognitive Examination–Revised, Consortium to Establish a Registry for Alzheimer’s Disease, MoCA 8.1, Qmci, and Memory Alternation Test are reported to have similar diagnostic accuracy [61,88]. The HCPs participating in this study reported using the MoCA 8.1 as their primary screening tool for MCI along with other assessments such as the MMSE and Trail Making Test parts A and B.

Recent research highlights the growing use of digital tools [51,89,90], mobile technology [91,92], virtual reality [93,94], and artificial intelligence [95] to improve early identification of MCI. Demeyere et al [51] developed the tablet-based, 10-item Oxford Cognitive Screen–Plus to detect slight changes in cognitive impairment across 5 domains of cognition (memory, attention, number, praxis, and language), which has been validated among neurologically healthy older adults. Statsenko et al [96] have explored improvement of the predictive capabilities of tests using artificial intelligence. Similarly, there is an emerging focus on the use of machine learning techniques to detect dementia leveraging routinely collected clinical data [97,98]. This progression signifies a shift toward more technologically advanced, efficient, and potentially more accurate diagnostic approaches in the detection of MCI.

Whatever the modality, screening tools should be quick to administer, demonstrate consistent results over time and between different evaluators, cover all major cognitive areas, and be straightforward to both administer and interpret [99]. However, highly sensitive tests such as those suggested for screening carry a significant risk of false-positive diagnoses [15]. Given the high potential for harm of false positives, it is important to validate the psychometric properties of screening tests across different populations and understand how factors such as age and education can influence the results [99].

Our study did not assess the face validity of the SCREEN, but participating occupational therapists were comfortable with the test regimen. Nonetheless, the research team noted the absence of verbal fluency and memory tests in the SCREEN, both of which McDonnell et al [100] identified as being more sensitive to the more commonly seen amnesic MCI. Two of the most sensitive tools for MCI screening, the MoCA 8.1 [76] and Qmci [25], assess memory, language, and verbal skills, and tests of verbal fluency and logical memory have been shown to be particularly sensitive to early cognitive changes [77,78].

The constructs included in the SCREEN (Table 1) were selected based on a single non–peer-reviewed study [58] using the 360 and traumatic brain injury data (N=188) that identified the constructs as predictive of brain injury. The absence of tasks that measure verbal fluency or logical memory in the SCREEN appears to weaken claims of construct validity. The principal component analysis of the SCREEN assessment identified 2 components accounting for 52.12% of the total variance. The first component was strongly associated with abstract reasoning, constructive ability, and divided attention, whereas the second was primarily influenced by visual-spatial abilities. This indicates that constructs related to perception, attention, and memory are central to the SCREEN scores.

The SCREEN’s binary outcome (healthy or unhealthy) created by the research team was based on comparisons with the Qmci. However, the method of identifying *areas of challenge* in the SCREEN by comparing the individual’s mean score on each of the 7 tasks with the mean scores of a global or filtered cohort in the LBB introduces potential biases or errors. These could arise from a surge in additions to the LBB from patients with specific characteristics, self-selection of participants, poorly trained SCREEN administrators, inclusion of nonstandard test results, underuse of appropriate filters, and underreporting of clinical conditions or factors such as socioeconomic status that impact performance in standardized cognitive tests.

The proprietary method of analyzing and reporting SCREEN results complicates traditional sensitivity and specificity measurement. Our testing indicated a sensitivity of 63.25% and specificity of 74.07% for identifying healthy (those without MCI) and unhealthy (those with MCI) individuals. The SCREEN’s Cronbach α=.63, slightly below the threshold for clinical instruments, and reliability scores that were lower than the ideal standards suggest a higher-than-acceptable level of random measurement error in its constructs. The lower reliability may also stem from an inadequate sample size or a limited number of scale items.

The SCREEN’s results are less favorable compared to those of other digital MCI screening tools that similarly enable evaluation of specific cognitive domains but also provide validated, norm-referenced cutoff scores and methods for cumulative scoring in clinical settings (Oxford Cognitive Screen–Plus) [51] or of validated MCI screening tools used in primary care (eg, MoCA 8.1, Qmci, and MMSE) [51,87]. The SCREEN’s unique scoring algorithm and the dynamic denominator in data analysis necessitate caution in comparing these results to those of other tools with fixed scoring algorithms and known sensitivities [101,102]. We found the SCREEN to have lower-than-expected internal reliability, suggesting significant random measurement error. Test-retest reliability was weak for the healthy or unhealthy outcome but stronger for overall activity scores between tests. The variability in identifying areas of challenge could relate to technological difficulties or variability from comparisons with a growing database of test results.

Potential reasons for older adults’ poorer scores on timed tests include the impact of sensorimotor decline on touch screen sensation and reaction time [38,103], anxiety related to taking a computer-enabled test [104-106], or the anticipated consequences of a negative outcome [107]. However, these effects were unlikely to have influenced the results of this study. Practice effects were observed [29,108], but the SCREEN’s novelty suggests that familiarity is not gained through prepreparation or word of mouth as this sample was self-selected and not randomized. Future research might also explore the impact of digital literacy and cultural differences in the interpretation of software constructs or icons on MCI screening in a randomized, older adult sample.

### Limitations

This study had methodological limitations that warrant attention. The small sample size and the demographic distribution of the 147 participants aged ≥55 years, with most (96/147, 65.3%) being female and well educated, limits the generalizability of the findings to different populations. The study’s design, aiming to explore the sensitivity of the SCREEN for early detection of MCI, necessitated the exclusion of individuals with a previous diagnosis of MCI or dementia. This exclusion criterion might have impacted the study’s ability to thoroughly assess the SCREEN’s effectiveness in a more varied clinical context. The requirement for participants to read and comprehend English introduced another limitation to our study. This criterion potentially limited the SCREEN tool’s applicability across diverse linguistic backgrounds as individuals with language-based impairments or those not proficient in English may face challenges in completing the assessment [51]. Such limitations could impact the generalizability of our findings to non–English-speaking populations or to those with language impairments, underscoring the need for further research to evaluate the SCREEN tool’s effectiveness in broader clinical and linguistic contexts.

Financial constraints played a role in limiting the study’s scope. Due to funding limitations, it was not possible to include specialist assessments and a battery of neuropsychiatric tests generally considered the *gold standard* to confirm or rule out an MCI diagnosis. Therefore, the study relied on differential verification through 2 imperfect reference standards: a comparison with the Qmci (the tool with the highest published sensitivity to MCI in 2019, when the study was designed) and the clinical judgment of the administering HCP, particularly in decisions regarding referrals for further clinical assessment. Furthermore, while an economic feasibility assessment was considered, the research team determined that it should follow, not precede, an evaluation of the SCREEN’s validity and reliability.

The proprietary nature of the algorithm used for scoring the SCREEN posed another challenge. Without access to this algorithm, the research team had to use a novel comparative statistical approach, coding patient results into a binary variable: healthy (SCREEN=no areas of challenge OR Qmci≥67 out of 100) or unhealthy (SCREEN=one or more areas of challenge OR Qmci<67 out of 100). This may have introduced a higher level of error into our statistical analysis. Furthermore, the process for determining areas of challenge on the SCREEN involves comparing a participant’s result to the existing SCREEN results in the LBB at the time of testing. By the end of this study, the LBB contained 632 SCREEN results for adults aged ≥55 years, with this study contributing 258 of those results. The remaining 366 original SCREEN results, 64% of which were completed by individuals who self-identified as having a preexisting diagnosis or conditions associated with cognitive impairment (eg, traumatic brain injury, concussion, or stroke), could have led to an overestimation of the means and SDs of the study participants’ results at the outset of the study.

Unlike other cognitive screening tools, the SCREEN allows for filtering of results to compare different patient cohorts in the LBB using criteria such as age and education. However, at this stage of the LBB’s development, using such filters can significantly reduce the reliability of the results due to a smaller comparator population (ie, the denominator used to calculate the mean and SD). This, in turn, affects the significance of the results. Moreover, the constantly changing LBB data set makes it challenging to meaningfully compare an individual’s results over time as the evolving denominator affects the accuracy and relevance of these comparisons. Finally, the significant improvement in SCREEN scores between the first and second visits suggests the presence of practice effects, which could have influenced the reliability and validity of the findings.

### Conclusions

In a primary care setting, where MCI screening tools are essential and recommended for those with concerns [85], certain criteria are paramount: time efficiency, ease of administration, and robust psychometric properties [82]. Our analysis of the BrainFx SCREEN suggests that, despite its innovative approach and digital delivery, it currently falls short in meeting these criteria. The SCREEN’s comparatively longer administration time and lower-than-expected reliability scores suggest that it may not be the most effective tool for MCI screening of older adults in a primary care setting at this time.

It is important to note that, in the wake of the COVID-19 pandemic, and with an aging population living and aging by design or necessity in a community setting, there is growing interest in digital solutions, including web-based applications and platforms to both collect digital biomarkers and deliver cognitive training and other interventions [109,110]. However, new normative standards are required when adapting cognitive tests to digital formats [92] as the change in medium can significantly impact test performance and results interpretation. Therefore, we recommend caution when interpreting our study results and encourage continued research and refinement of tools such as the SCREEN. This ongoing process will ensure that current and future MCI screening tools are effective, reliable, and relevant in meeting the needs of our aging population, particularly in primary care settings where early detection and intervention are key.

## Abbreviations

ADL: activities of daily living
FHT: Family Health Team
GAS: Geriatric Anxiety Scale
GAS-10: Geriatric Anxiety Scale–10
HCP: health care provider
ICC: intraclass correlation coefficient
LBB: Living Brain Bank
MCI: mild cognitive impairment
MMSE: Mini-Mental State Examination
MoCA: Montreal Cognitive Assessment
Qmci: Quick Mild Cognitive Impairment

## Data Availability

The data sets generated during and analyzed during this study are available from the corresponding author on reasonable request.
